# Moving data off the shelf and into action: an intervention to improve data-informed decision making in Côte d'Ivoire

**DOI:** 10.3402/gha.v7.25035

**Published:** 2014-10-01

**Authors:** Tara Nutley, Léontine Gnassou, Moussa Traore, Abitche Edwige Bosso, Stephanie Mullen

**Affiliations:** 1 MEASURE Evaluation, Futures Group, Chapel Hill, NC, USA; 2 MEASURE Evaluation, John Snow, Inc., Abidjan, Côte d'Ivoire; 3 MEASURE Evaluation, John Snow, Inc., Rosslyn, VA, USA

**Keywords:** data use, data-informed decision making, HIV and AIDS, district level, routine health information systems, guidance

## Abstract

**Background:**

Improving a health system requires data, but too often they are unused or under-used by decision makers. Without interventions to improve the use of data in decision making, health systems cannot meet the needs of the populations they serve. In 2008, in Côte d'Ivoire, data were largely unused in health decision-making processes.

**Objective:**

To implement and evaluate an intervention to improve the use of data in decision making in Cote d'Ivoire.

**Design:**

From 2008 to 2012, Cote d'Ivoire sought to improve the use of national health data through an intervention that broadens participation in and builds links between data collection and decision-making processes; identifies information needs; improves data quality; builds capacity to analyze, synthesize, and interpret data; and develops policies to support data use. To assess the results, a Performance of Routine Information System Management Assessment was conducted before and after the intervention using a combination of purposeful and random sampling. In 2008, the sample consisted of the central level, 12 districts, and 119 facilities, and in 2012, the sample consisted of the central level, 20 districts, and 190 health facilities. To assess data use, we developed dichotomous indicators: discussions of analysis findings, decisions taken based on the analysis, and decisions referred to upper management for action. We aggregated the indicators to generate a composite, continuous index of data use.

**Results:**

From 2008 to 2012, the district data-use score increased from 40 to 70%; the facility score remained the same – 38%. The central score is not reported, because of a methodological difference in the two assessments.

**Conclusions:**

The intervention improved the use of data in decision making at the district level in Côte d'Ivoire. This study provides an example of, and guidance for, implementing a large-scale intervention to improve data-informed decision making.

According to the World Health Organization (WHO), a health system has six sectors: the health workforce; health services; health financing; governance and leadership; medical products, vaccines, and technologies; and health information (1). Of these, high-quality and timely data from a health information system (HIS) are a health system's foundation, because they inform decision making in each of the other five sectors (2). Many countries and international donors have committed to strengthen the quality, relevance, and comprehensiveness of HIS data (3). Their goal is data-informed decision making: the consideration of data during program monitoring, review, planning, and improvement; advocacy; and policy development and review (4). Too often, though, data are ignored or under-used, and data-informed decision making does not occur (5). Just because high-quality data^1^ are collected does not mean they will be used (2). Intervention may be necessary (6, 7).

This paper describes one such intervention, implemented with Côte d'Ivoire's Ministère de la Santé et de l'Hygiène Publique (MSHP), and the improvements that resulted in the quality, availability, and use of data from the national routine health information system (RHIS)^2^ (8). The intervention focused on three interrelated domains that affect the performance and use of RHIS:

1. Technical: that is, systems or processes to collect, review, and discuss data.
2. Behavioral: that is, how users handle data to solve problems and improve programs.
3. Organizational: that is, the structure and processes of the organizations that need the data.

These domains are part of the Performance of Routine Information System Management (PRISM) framework, as defined by Aqil et al. (9). PRISM offers a broad perspective on RHIS, by proposing that the technical, behavioral, and organizational domains mediate the success of such a system in measurable ways. Few projects reported in the literature address all three domains of the PRISM framework. This manuscript contributes to the literature, by describing a comprehensive intervention that does address them all, by linking data to decision-making processes, identifying the information needs of decision makers, building the capacity of users to analyze and interpret data, and strengthening the capacity of organizations to support and sustain data-use activities (Fig. 1) (6, 7).

**Fig. 1:**
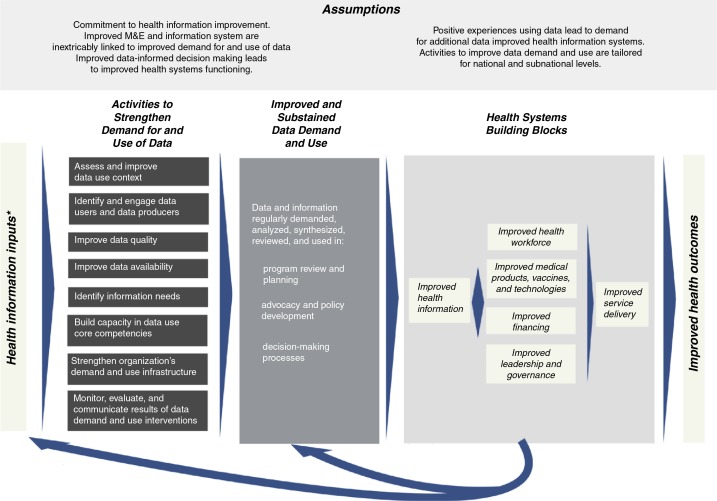
Conceptual framework: intervention to strengthen the use of health data in decision making (1, 7). *Defined as processes by Health Metrics Network. ^†^The data demand and use approach broadly defines an organization as a division of the ministry of health at the national, state, or district level; a specific program within the ministry; or non-governmental organization or program.

## History of routine information systems in Côte d'Ivoire

The MSHP and partners implemented a national HIS called the Système d'Information de Gestion (SIG) in 1995. The SIG collected routine data from primary health care throughout the country using standard, paper-based data collection and management tools (facility registers and patient records). Data were transferred in monthly, quarterly, and annual reports to the district, regional, and central offices. A computerized data management application facilitated the process.

Between 1995 and 2007, the MSHP and its partners implemented efforts to improve the SIG, assigning priority to data quality, indicator harmonization, and standardization; database harmonization; and the production of SIG reporting tools. In 2007, the MSHP rolled out the revised SIG and data management application nationwide. Despite the improvements, the system's implementation suffered from political instability and high staff turnover in the ministry of health. Also, challenges in identifying and committing resources to maintain the system contributed to low stakeholder buy-in. As a result, data were still largely unavailable for decision making and those data that were available were not always considered.

## Design: a comprehensive intervention to improve data use

The intervention to strengthen the use of health data in decision making that Cote d'Ivoire used consists of eight activities that are central to the use of data in decision making:

1. Assess and improve the data-use context
2. Engage data users and data producers
3. Improve data quality
4. Improve data availability
5. Identify information needs
6. Build capacity in data-use core competencies
7. Strengthen the organization's data-use infrastructure
8. Monitor and evaluate data-use interventions

A logic model (Fig. 1) (6) maps out how the intervention's inputs and activities should influence the outputs and eventual outcome of regular data use in program review, planning, advocacy, policy development, and other decision-making processes. The intervention is unique, because it recommends improvements in the three domains of routine information system performance (technical, behavioral, and organizational) and recognizes that usually no single activity is sufficient to achieve lasting improvements in data use. The intervention, although rooted in the published literature (4, 9–12) , had not been implemented in its entirety, nor had it been formally evaluated until the experience in Côte d'Ivoire. The sections below describe how the country undertook each of the intervention's activities over the years between 2008 and 2012. This paper reports primarily on the activities implemented to improve the data systems and processes for HIV data.

### Activity: assess and improve the data-use context

Côte d'Ivoire began, in 2008, by conducting a PRISM assessment to understand fully why routine health information was little-used (8). Four survey instruments were implemented to assess the RHIS's performance and processes as well as the technical, behavioral, and organizational determinants that mediate the performance of the system. The variables that PRISM investigates are the quality of data (accuracy, completeness, and timeliness), data analysis practices, and the use of information. The PRISM assessment in Côte d'Ivoire looked specifically at the data from HIV testing, maternal health, child health, and malaria services. The ministry used the assessment's results to develop an RHIS strengthening plan rooted in improving the use of data in decision making in all health programs (see methods section for more information on the PRISM methodology).

#### Activity: engage data producers and data users

We define data producers as researchers and also those who design and manage information systems, such as monitoring and evaluation (M&E) specialists. Data users are those who use data to develop and improve programs and policies, such as program managers, policy makers, and program directors. The closer the relationship between data producers and users, the greater is the value that both groups place on data. When the perceived value of data increases, the sense of ownership of data increases, as well, and programs improve (10, 13–16) .

Multiple activities were undertaken to ensure that data users and producers participated in the national effort to improve the SIG. The MSHP began by facilitating and financially supporting two series of quarterly forums to coordinate strategic information. The first was a national health management information system (HMIS) working group. Its members, M&E and program staff from the government, and partner organizations met to share information, harmonize and validate national data collection forms and applications, coordinate HMIS activities, and exchange best practices in M&E and data use. The other series of forums brought together regional and district program managers and data managers from 6 of the country's 19 regions to discuss data quality and data analysis findings and to develop data quality and data-use plans.

To further encourage data users and producers to collaborate, a meeting was convened that included decision makers, program managers, and M&E specialists at the national level from MSHP and the three other ministries with HIV and AIDS programs. Prior to this meeting, the four ministries had never coordinated HIV programming or discussed data systems and use.

During the meeting, each ministry identified barriers to data-informed decision making and blockages in the flow of information and solutions to overcome them. Each ministry made its own action plan for implementation. The ministries also made a joint action plan to overcome barriers they share. Although political instability in Côte d'Ivoire prevented implementation of some recommendations in the action plans, this initiative engendered collaboration among the ministries and united them to work jointly on improving data use.

### Activity: improve data quality

Data users need to know that they can trust the information on which they base their decisions. When the quality of data is low, demand for data decreases, data-informed decision making does not occur, and program efficiency and effectiveness suffer (4, 16, 17).

The 2008 PRISM assessment and the four ministries at the 2010 data-use workshop identified poor data quality as a primary barrier to the use of data in decision making. To address this barrier, the MSHP established a tiered system of audits to assess data quality. It followed up by developing and implementing action plans to address the problems that the audits revealed.

The ministry's data quality assessment system consisted of data quality audits (DQAs) at the national level and routine data quality assessments (RDQAs) at the regional level. DQAs are primarily an external audit process that focuses on uncovering hidden problems in collection, aggregation, and transmission of priority indicator data. RDQAs are a simplified version of the DQA; they are self-administered and focus on verifying the quality of reported data and on assessing and improving the underlying data management and reporting systems (18).

At the national level, the MSHP developed terms of reference for the RDQA and a national protocol for a DQA. In 2011, a DQA was implemented in all HIV care and treatment sites in Côte d'Ivoire. The result was an action plan and budget for improving data quality in HIV care and treatment programs. At the subnational level, key stakeholders were trained to use the RDQA and institutionalize the use of the RDQA tool as part of national supervision guidelines. A standardized *Data Management Procedures Manual* was also developed to support this process. The four ministries tasked with HIV and AIDS programs are now regularly implementing RDQAs.

### Activity: improve data availability

To inform decision making, data must be available the moment they are needed (11). Data also need to be synthesized in formats that users can understand and explain to others (19).

Results of previous HIS assessments in Côte d'Ivoire found weaknesses in the availability of health data at all levels of the system (20). Multiple, yet unlinked, HIV databases inhibited easy access to available data. This resulted in delayed data aggregation, synthesis, and dissemination. For example, the 2002 *National Annual Health Report* describing the health situation in Côte d'Ivoire was based on 1999 data and was not published until 2003.

In response, ACONDA VS and the Institut de Santé Publique et de Developpement developed an electronic medical record called Systeme d'Information et de Gestion du Dossier Electronique du Patient (SIGDEP), which became available for use in 2010. SIGDEP manages patient data from HIV care and treatment sites as a single national system. SIGDEP data can be used along with RHIS data to generate monthly summary reports as well as more granular analyses of care and treatment information organized by patient cohorts, reports on the provision and results of voluntary counseling and testing, and reports on the management of antiretroviral therapy and other drugs. SIGDEP also generates monthly reports at the facility/site level. SIGDEP gives clinical providers and managers access to relevant data for decision making. For example, program managers are now able to estimate the national need for antiretroviral and opportunistic infection drugs.

### Activity: identify information needs

The quantity of data available to decision makers is often overwhelming. When decision makers receive only the data they need to know to run health programs, their use of data improves (14, 15, 21). At the previously described regional meetings, ministries identified the RHIS data they needed and established a data-use plan.^3^ The data-use plan allowed the regional representatives to identify specific questions relevant to their regions, link the questions to existing data, and make recommendations to address issues that emerged in their data review. Questions that could not be answered with data were also entered on the data-use plan and a timeline was created with deadlines for acquiring the data. The timeline detailed the roles and responsibilities of those responsible, and specified follow-up activities to document decisions made when the missing data were supplied. Such meetings are a promising practice for institutionalizing a process to regularly target key information needs.

### Activity: build data-use core competencies

For data to be a routine part of decision making, people at all levels of the health system must have the skills to analyze, interpret, synthesize, present, and use data (6).

The 2008 PRISM assessment found these skills to be weak among health professionals in Côte d'Ivoire (8). To correct this problem, these skills, along with traditional M&E practices, were added to both in-service and pre-service curricula of three local training institutions. Government health workers, data managers, and clinicians were trained, as well as other health professionals employed by non-governmental organizations. As of 2014, 479 people were trained.

Although PRISM does not reflect results of activities implemented after 2011, the ongoing expanded curricula at these four institutions illustrate a national commitment to creating sustained data-use capacity.

### Activity: strengthen organizations’ data-use infrastructure

Without sufficient staff to lead, implement, and supervise an M&E system, data will not be available for use in decision making.

An insufficient number of skilled M&E professionals, particularly at the regional level, posed challenges for further RHIS strengthening. In 2007, the staffing gap prompted the MSHP to create new regional M&E units to oversee data management, conduct regular M&E supervision, transmit data to the central level, and lead data-informed decision making. Furthermore, in 2012, the MSHP issued a memo to all health regions and districts mandating the creation of a ‘director of monitoring and evaluation’ position, to ensure that M&E and data-informed decision making would have higher priority.

In an effort to develop leadership of M&E systems and use of data they generate, the four ministries involved in HIV programming participated in a virtual leadership development program. The program produced leaders who are building a culture of data use, by advocating and fundraising for high-quality and timely national HIV and AIDS data. They have facilitated the development of a common vision for data-informed decision making.

For people to use data in decision making in a sustainable way, their organizations need to support them with clear processes and systems that help them to undertake data-use tasks (9, 22). A number of resources provided organizational support for staff tasked with data-use responsibilities. These were a set of national supervision guidelines (which included data-quality checks), the *Data Management Procedures Manual*, the *National DQA Protocol*, and terms of reference for RDQA. These supports provided guidance for task implementation. Moreover, all the improvements that were implemented to ensure adequate human resources to support M&E and the data-use intervention also strengthened the MSHP's infrastructure, so that the ministry can sustain the data-use intervention.

### Activity: monitor and evaluate the intervention

Regular use of data in decision making generates demand for quality data and the reinforcement of data-informed decision making processes (4). The evaluation of data-use interventions and the communication of successful interventions build the knowledge base that supports future investments in interventions to strengthen data use.

To understand the effect of the intervention on data-informed decision making, we implemented a second PRISM assessment in 2012 – 4 years after the first PRISM assessment (23).

## Design: methods

The 2012 PRISM consisted of purposefully selecting the two regions of Lagunes and randomly selecting eight other regions (for a total of 10 out of the 20 regions in Côte d’ Ivoire). Four of the regions surveyed participated in the data-use intervention. In each region, two districts were selected – the district with the regional headquarters plus one other at random, for a total of 20 districts. At the health facility level, all central university hospitals, regional hospitals, and general hospitals were included in the sample. In addition, in each district, nine other health facilities were randomly selected from the list of hospitals that reported to the MSHP.

The 2008 and 2012 PRISM assessments used the same sampling approach, data collection tools, and analysis plan, but the sample sizes differed. The 2012 assessment covered four additional regions (an increase from 6 to 10) in order to create a more representative sample. This also resulted in an increased number of facilities. Moreover, the 2008 assessment did not measure data use at the central level. (See the text box for the data collection tools used in both assessments and Table 1 for a summary of the sample in the 2008 and 2012 assessments.)

**Table 1:** PRISM methodology summary for 2008 and 2012

	2008 PRISM	2012 PRISM
Location	12 districts 6 health regions 119 health facilities	Central – DIPE 20 districts 10 health regions 190 health facilities
Facility register and report review	119 facilities	190 facilities
District database review	12 districts	20 districts
Region database review	Not available	10 regions
Observation at region, district, and facility levels	Information displays and meeting reports	Information displays and meeting reports
Interview	132 individuals^a^	221 individuals^b^
Self-administered questionnaire (OBAT)	143 individuals	342 individuals
Data entry	Microsoft Excel	PRISM DEAT^c^
Data analysis	Microsoft Excel and SPSS^d^	PRISM DEAT

a District chief medical officers and chiefs of the epidemiological surveillance center. Health facility managers and data administrators.

b District and region chief medical officers, chiefs of the epidemiological surveillance center, chiefs of monitoring and evaluation. Health facility managers and data administrators.

c Data entry and analysis tool.

d Statistical Package for the Social Sciences.

PRISM Data Collection Tools/Approaches

1. *Performance Diagnostic Tool:* Determines the overall RHIS performance. Assesses the use of information for problem identification and solving, decision making, resource mobilization, and monitoring
2. *RHIS overview and Facility/Office Checklist:* Examines technical determinants, such as the structure and design of existing information systems, information flow, and the interaction between different information systems
3. *Management Assessment Tool:* Measures different RHIS management functions including governance, planning, training, supervision, use of performance improvement tools, and financial resources
4. *Organizational and Behavioral Assessment Tool*: Identifies behavioral and organizational factors that affect RHIS performance, including data demand, motivation, confidence level, task competence, and problem-solving skills
5. Database review
6. Facility data display, report, and register review

The 2012 assessment was conducted at the central, regional, district, and facility levels. Ten health regions, 20 health districts, and 190 health facilities participated in the assessment. Analyses were conducted using Microsoft Excel in 2008. In 2012, a tool was developed using Microsoft Excel and Access to facilitate data entry, management, and analysis.

PRISM measures multiple components of RHIS performance. However, for our purposes here, we discuss only data quality, data availability, and the use of information. We compare the 2012 data for these measures to the 2008 data to illustrate improvements in these domains.

## Results and discussion

### Data quality

PRISM's measures of data quality are completeness and accuracy. At the district and regional levels, completeness refers to the percentage of all reports that are transmitted on time. At the facility level, completeness refers to the percentage of the items in the report that are complete. Accuracy is the percentage of figures reported that match figures aggregated from primary data sources.

**Fig. 2:**
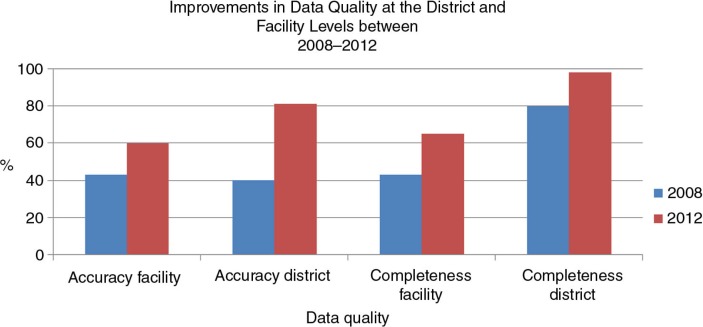
Improvements in data quality at the district and facility levels between 2008 and 2012.

To measure these elements, we consulted monthly reports and primary data sources and discovered the following improvements from 2008 to 2012:

1. Data accuracy: facility level from 43 to 60%; district level from 40 to 81%
2. Data completeness: facility level from 43 to 65%; district level from 80 to 98% (Fig. 2)

The assessment also revealed a notable improvement in data quality as assessed during supervisory visits. This indicator increased at the facility level from 36% in 2008 to 90% in 2012.

### Data availability

Data availability is defined as data that are synthesized into formats that are understandable, actively communicated to potential users, and easy to access by those who need them (6). The 2012 PRISM assessment showed the following improvements in data availability from 2008 to 2012:

1. Information feedback after supervisory visits: facility level from 7 to 29%
2. Availability of data summary reports: facility level from 12 to 38%; district level from 29 to 65%

In addition, SIGDEP, which manages data for 233 HIV sites, has facilitated the availability of data on HIV patients tracking antiretroviral therapy through data dashboards, which quickly synthesize and present data in formats that are more accessible for decision making. As a result, the MSHP is now able to review data midyear and produce the national HIV report in a timely manner. Data summary reports, tailored to specific regions, have also been produced and are currently being distributed in a few regions.

### Data use

To determine the extent to which data were used in decision making, PRISM uses a series of dichotomous indicators: whether RHIS information was discussed in staff meetings, whether decisions evolved from these discussions, and whether the decisions were referred to upper management for action. These indicators were aggregated to generate a composite, continuous index of the use of RHIS information (24). This approach gives equal weight to each of the indicators used in the index.

The improvement in these indicators from 2008 to 2012 was as follows:

1. Discussion of RHIS analyses: district level from 40 to 82%; facility level from 34 to 42%
2. Decisions taken: district level from 25 to 73%; facility level from 31 to 39%
3. Decisions referred to upper management: district level from 43 to 64%; facility level from 31 to 37%

**Fig. 3:**
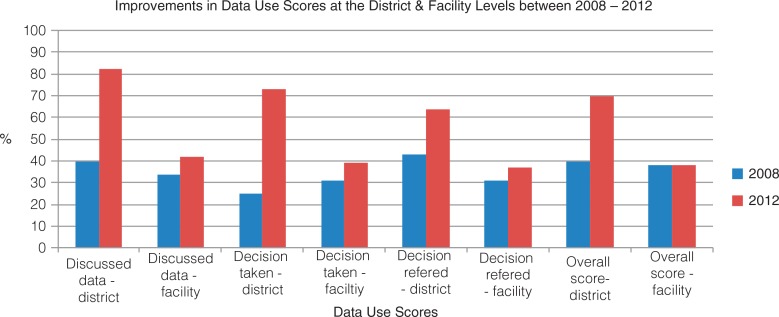
Improvements in data-use scores at the district and facility levels between 2008 and 2012.

The global data-use score at the district level increased from 44% in 2008 to 70% in 2012 (Fig. 3). The data-use score at the facility level remained the same – 38%. Any changes that may have occurred in the data-use scores at the regional and central levels are not reported here, because these measures were not taken in 2008. In 2012, the data-use score at the regional level was 50%; at the central level it was 100%.

The 2012 PRISM assessment also found that, during the 3 months preceding the survey, the MSHP had organized and officially recorded eight meetings to review data. Official records of the meetings confirm that participants discussed data and used them to make decisions.

### PRISM results discussion

These results suggest that the quality, availability, and use of HIV data improved during the time that the comprehensive data-use intervention was being implemented.

The most important finding from the two PRISM assessments is the increase in the use of data in decision making at the district level from 40% in 2008 to 70% in 2012. This suggests that the implementation of the data-use intervention succeeded in its intended mission.

The lack of increases at the facility level between 2008 and 2012 is explained by the focus on data quality, which was not combined with the other complementary activities critical to improving data use overall.

In addition, the 2012 assessment found a 100% data-use score at the central level. Although we are unable to quantitatively determine the effect of the intervention on this measure, we posit that the intervention contributed to improvements in the use of data at the central level. Prior to 2008, data were largely unavailable for decision making. The finding that eight data-review sessions had been held during the 3 months preceding the 2012 PRISM suggests that an active review and discussion of data took place. We theorize that the improvements in data quality and availability enabled the MSHP to access data more easily than in the past and to place more confidence in the data. Official records of the meetings (collected as part of the performance diagnostic tool) confirm that participants discussed data and used them to make decisions.

The greatest improvement in data quality (data completeness and accuracy, as defined by PRISM) was the twofold increase in data accuracy at the district level. Data accuracy also increased at the facility level, from 43% in 2008 to 60% in 2012. Data completeness increased approximately 20 percentage points for both the facility and district levels. The implementation of DQAs and RDQAs at the national and subnational levels and the training of a cadre of professionals to teach others to conduct these assessments most likely contributed to these results. Moreover, in an effort to ensure standardization of the DQA and RDQA, the MSHP developed national terms of reference and implementation guidelines. Assuring data quality became a regular job function of facility supervisors. The MSHP also developed national guidelines for supervisors, in order to institutionalize the standardized data management procedures for health and non-health data. These efforts not only contributed to the production of a regular supply of quality data but also strengthened the ministry's infrastructure to support and sustain the data-use intervention. The standardized DQA and RDQA protocols, tools, and guidelines make the trend of improving data quality sustainable. New positions were created and capacity was built to implement and manage future data-quality activities. The commitment of local, pre-service training institutions to train new cohorts of professionals in data use also promises to sustain the intervention long term.

To measure data availability, PRISM assessed improvements in information feedback to the facility level after supervisory visits and the availability of data summary reports at the facility and district levels. The improvements measured in these areas were substantial. Following the MSHP's new national guidelines, supervisors at the district level share synthesized data with facilities. This enables front-line service providers to understand how their work contributes to district-level targets and objectives. Even though the increase in information feedback after supervisory visits was low (from 7 to 29%), it shows progress in the availability of data subnationally. The increases seen in the availability of data summary reports (from 12 to 38% at the facility level and from 29 to 65% at the district level) are attributable to the SIGDEP database's capacity to generate customized reports that synthesize data and to the generation of tailored communication products for national- and district-level use. Also, because data were no longer fragmented into multiple data sets, data users were able to find and access the data they needed to monitor their programs and conduct analyses. The addition of SIGDEP to the M&E system improved the information technology infrastructure and improved data access.

### Observed results

The authors posit that other activity areas outlined in the data-use intervention that PRISM did not measure contributed to increases in data quality, data availability, and data use. For example, PRISM does not measure improvements in individual capacity to use data, increases in engagement of data users and data producers, increases in the identification of information needs, or the improved ability of an organization to support data use. Even so, we can describe improvements in these areas and plausibly connect their contributions to the improvements that PRISM did measure.

We saw multiple instances of improvements in the areas of engagement of data users and producers to work together to use data and identify information needs in Côte d'Ivoire. Two types of quarterly strategic coordination meetings were established. The first, at the national level, addresses M&E in general and data use specifically, and the second, at the regional level, allows districts to use tools to review data and develop data-use plans. These meetings allow data producers and users to jointly discuss and identify key programmatic questions, link these questions to available data, and analyze the data to come up with answers. This gives producers and users a shared sense of ownership of data, inclining them to move decisions forward.

The courses on data quality and data-informed decision making at national training institutions and universities contributed to increased capacity to use data and the increased use of data. The courses on M&E, data quality, and data use were first offered in 2010. The institutions offering the courses contributed new employees to the national public health system and the MSHP specifically. In addition, two of the training institutions offered the courses to MSHP M&E staff and clinical staff, in addition to students, who immediately went back to their places of employment with their new knowledge.

The last step of the data-use intervention was the institutionalization of data in decision making. Successes in this area were the development of national guidelines, protocols, and other guidance on data use; the regular convening of national and subnational working groups dedicated to data use; and the creation of new jobs to oversee data-use activities. In addition, the work with the local training institutions will sustain the country's capacity for data use as graduates enter the workforce better equipped to use data in decision making.

Improvement in the use of data in decision making has implications for significantly improving policy formulation and the future delivery of health services in Côte d'Ivoire. For example, data from the Demographic and Health Survey conducted in Côte d'Ivoire in 2011–2012 found HIV prevalence to be higher among women (4.6%) than among men (2.9%) (25). In response to this, the MSHP prioritized improvements in programs to prevent HIV among women. The improved quality, availability, and use of RHIS data will now allow the MSHP to regularly review program performance and formulate policies to continuously meet the needs of women at risk of HIV.

### Limitations

#### Our methods have three main limitations

First is the assumption that the data-use intervention was responsible for all of the measured and observed changes in data quality and data use. From 2009 to 2011, the Programme National de Prise en Charge Médicale des Personnes vivant avec le VIH, le Projet de l'Amélioration des Soins de Santé, and other stakeholders embarked on a collaborative project to improve provision of HIV care and treatment services. The project overlapped in four regions where we implemented the data-use intervention. The project's focus was to improve HIV patient tracking and follow-up, increase availability and competence of health workers, and improve service organization. As part of the project, coaches helped teams to improve the quality of facility-level HIV information and gather baseline data. They also coached the teams to analyze their data and discuss problems and improvements in HIV services. Teams also monitored a common set of core indicators to assess improvements in quality. It is possible that this model of data review, discussion, and action contributed to the improvements in data use as measured by PRISM.

Second, although PRISM provides a reliable framework for assessing an RHIS's performance, it suffers from methodological limitations (26). Moreover, PRISM does not assess changes in four of the activity areas outlined in the comprehensive intervention: engage data users and data producers, identify information needs, build data-use capacities, and strengthen an organization's data-use infrastructure. Therefore, quantitative improvements in these areas were not documented.

Third, the relative contribution of each activity area of the intervention to the data-use outcome is not well understood. Because PRISM does not measure three of the activity areas that affect decision making, we do not understand the magnitude or relative strength of their relationships on the outcome of interest.

As more interventions are designed and applied to improve the use of health data in decision making, they need to be evaluated. PRISM provides a reliable framework within which to measure the primary outcomes of an intervention. However, more rigorous evaluations need to be implemented to determine the relative importance and intensity of each one of the intervention's activities. Moreover, better measures of the use of data in decision making are also needed.

## Conclusions

Information systems are undoubtedly valuable in improving the delivery of health services, health systems, and, ultimately, health outcomes. Insufficient demand for and use of data limit a health system's capacity to respond to priority needs at every level.

This article describes how comprehensive data-use intervention addresses behavioral, technical, and organizational constraints to data use. Such an intervention puts efforts to improve programs on a solid foundation, and positions the use of data in decision making to become a sustainable practice. Many published works discuss the use of data in decision making, but few provide guidance to surmount the three domains of constraints. Thus, this report fills a significant gap in the published literature.

## Footnotes

1 The dimensions of data quality as defined by the DQA Tool Guidelines for Implementation are accuracy, reliability, precision, completeness, timeliness, integrity, and confidentiality. (Source: MEASURE Evaluation. DQA tool guidelines for implementation.)

## Footnotes

2 Health data and information come from a variety of sources. There are population-based sources, such as censuses, vital registration, and household surveys. And there are institution-based sources, such as health facility surveys and records and patient records. Routine health information systems (RHIS) collect data from health facilities that are recorded as part of the facilities’ daily operations. (Source: Health Metrics Network. Framework and Standards for Country Health Information Systems. Health Metrics Network: World Health Organization, 2008.)

## Footnotes

1 The data use plan was based on a management tool developed by MEASURE Evaluation, available at http://www.cpc.unc.edu/measure/publications/ms-11-46-b.
